# Draft Genome Sequence of a Marine Photoferrotrophic Bacterium, Rhodovulum robiginosum DSM 12329^T^

**DOI:** 10.1128/MRA.01684-18

**Published:** 2019-02-21

**Authors:** Dinesh Gupta, Michael S. Guzman, Arpita Bose

**Affiliations:** a Department of Biology, Washington University in St. Louis, St. Louis, Missouri, USA; University of Maryland School of Medicine

## Abstract

Here, we report the draft genome sequence of Rhodovulum robiginosum DSM 12329^T^, an anoxygenic phototroph isolated from a marine sediment in the North Sea (Jadebusen, Germany). This is the first genome for a marine photoferrotrophic bacterium, and it provides a genetic basis to understand the mechanistic underpinnings of photoferrotrophy in future studies.

## ANNOUNCEMENT

Rhodovulum robiginosum DSM 12329^T^ is a Gram-negative purple bacterium, originally isolated from North Sea coastal sediment (Jadebusen, Germany), with ferrous iron as the sole electron donor for anoxygenic phototrophic growth (photoferrotrophy) (1). The isolation, phylogenetic analysis, and phototrophic growth characteristics of R. robiginosum DSM 12329^T^ have been previously published (1). This is one of two known photoferrotrophic bacteria belonging to the genus Rhodovulum (1). To better understand the mechanisms underlying photoferrotrophy, we sequenced and annotated the genome of R. robiginosum DSM 12329^T^.

R. robiginosum DSM 12329^T^ was obtained from the Leibniz-Institut DSMZ GmbH and grown anaerobically at 30°C for 5 days in 10 ml artificial seawater medium (1) supplemented with 10 mM acetate. Genomic DNA was prepared using a DNeasy blood and tissue kit following the manufacturer’s protocol (Qiagen, Dusseldorf, Germany). Sequencing libraries were prepared with a Nextera sample prep kit (Illumina Inc., San Diego, CA) following the manufacturer’s protocol. Paired-end (250 bp) sequencing was carried out to 100× coverage on a MiSeq instrument using v2 sequencing chemistry (Illumina Inc., San Diego, CA). Illumina sequencing resulted in 1,974,052 reads. The raw reads were quality filtered and adaptor trimmed using Trimmomatic version 0.38 with the default settings for paired-end reads (2). Processed reads were *de novo* assembled using SPAdes version 3.13.0 with the program’s default parameters (3). Genome assembly yielded 51 contigs. The total genome assembly size is 3,712,677 bp with an *N*_50_ value of 182,446 and a GC content of 67.5%. Functional analysis was performed using the Rapid Annotations using Subsystems Technology (RAST) server (4, 5) version 2.0 (http://rast.nmpdr.org/rast.cgi) via the RASTtk pipeline with the default settings (6). Sequences were annotated using the NCBI Prokaryotic Genome Annotation Pipeline (PGAP) (7).

NCBI PGAP identified a total of 3,653 genes, 3,557 protein-coding genes, 47 pseudogenes, and 43 tRNAs. Based on the RAST functional annotation, the genome includes genes for respiration, photosynthesis, nitrogen fixation, and the metabolism of aromatic compounds. The annotated genome encodes photosystem II and various reductases involved in anaerobic respiration, such as nitrate reductase and tetrathionate reductase. The genome also encodes a complete set of genes required for the assembly of [NiFe] hydrogenase. Genes encoding a putative type II protein secretion system, a type IV protein secretion system, an AttEFGH ABC transport system, and systems for cytoplasmic protein-translocation are found in the genome. The genome also contains a complete set of genes for cytochrome *c* maturation system I (*CcmABCDEFGH*).

Heme containing cytochrome *c* proteins are known to be involved in photoferrotrophy in related bacteria (8, 9). Genomewide searches for cytochrome *c* proteins containing the characteristic heme-binding motif (CXXCH) in the PGAP annotations yielded 40 proteins. This includes 30 monoheme, 8 diheme, 1 triheme, and 1 octaheme *c*-type cytochromes. This annotated genome sets the stage for future genetic studies of photoferrotrophy in R. robiginosum DSM 12329^T^ and improves our understanding of photoferrotrophic marine bacteria.

### Data availability.

This whole-genome shotgun project has been deposited in GenBank under the accession number RWGU00000000. Raw sequencing reads have been deposited in the NCBI Sequence Read Archive (SRA) under the accession number SRR8282973.
