# Correction for Gupta et al., “Draft Genome Sequence of a Marine Photoferrotrophic Bacterium, *Rhodovulum iodosum* DSM 12328^T^”

**DOI:** 10.1128/mra.00103-25

**Published:** 2025-07-29

**Authors:** Dinesh Gupta, Michael S. Guzman, Arpita Bose

## AUTHOR CORRECTION

Volume 8, no. 8, e01684-18, 2019, https://doi.org/10.1128/mra.01684-18. The title should read as given in this correction, and "*Rhodovulum robiginosum* DSM 12329^T^" should read "*Rhodovulum iodosum* DSM 12328^T^” throughout.

Page 1, paragraph 2, sentence 1: “*R. robiginosum* DSM 12329^T^ was obtained from the Leibniz-Institut DSMZ GmbH” should read “*R. iodosum* strain N1 DSM 12328^T^ (which is maintained as *Rhodovulum robiginosum* DSM 12329^T^ by the Leibniz-Institut DSMZ GmbH) was obtained from the Leibniz-Institut DSMZ GmbH.”

We obtained *Rhodovulum iodosum* strain N1 from the Leibniz-Institut DSMZ GmbH in 2018, where it is maintained as *Rhodovulum robiginosum* DSM 12329^T^. A potential swap of *Rhodovulum iodosum* DSM 12328^T^ and *Rhodovulum robiginosum* DSM 12329^T^ is evident in a recent study (1). We are publishing this correction to maintain the integrity of the scientific record and to emphasize the use of caution to the scientific community to confirm the strain they receive when conducting their research.
